# The Acceptability and Feasibility of a Preschool Intervention Targeting Motor, Social, and Emotional Development

**DOI:** 10.3389/fped.2020.00319

**Published:** 2020-07-10

**Authors:** Maeghan E. James, Chloe Bedard, Emily Bremer, John Cairney

**Affiliations:** ^1^Faculty of Kinesiology and Physical Education, University of Toronto, Toronto, ON, Canada; ^2^Department of Health Research Methods, Evidence, and Impact, McMaster University, Hamilton, ON, Canada; ^3^School of Human Movement and Nutrition Sciences, University of Queensland, St Lucia, QLD, Australia

**Keywords:** early childhood, fundamental movement skills, social–emotional learning, early intervention, child development

## Abstract

**Background:** Children and youth are facing three major challenges: (1) poor mental health, (2) physical inactivity, and (3) lack of school readiness. Fundamental movement skills (FMS) and social–emotional learning (SEL) are two developmental domains that are associated with each of these challenges. Currently, there is little focus on interventions that target both FMS and SEL. Thus, the purposes of this study were to: (1) examine the acceptability and feasibility of an FMS and SEL program (*Move 2 Smile*) and (2) assess the impact of *Move 2 Smile* on FMS and SEL in children.

**Methods:** An exploratory, pilot study using a within-subjects design was conducted. Descriptive statistics were computed to assess the acceptability and feasibility of the *Move 2 Smile* program. Changes in FMS and SEL were analyzed using a paired sample *t*-test. A focus group was conducted with parents to gain feedback after the program ended.

**Results:** Eleven children (four girls; *M*_age_ = 50.56 months, SD = 8.63) participated, with families attending 80% of the sessions. The children and parents rated the enjoyment of the program 4.1/5 and 4.7/5, respectively. The instructor rated the children's perceived enjoyment 4.6/5 and feasibility of the sessions 4.7/5. Parents engaged in the FMS take-home activities once per week and the SEL activities three times per week. The intervention had a non-significant small to medium effect on FMS (*d*_*z*_ = 0.42, *p* = 0.19), a significant large effect on social skills (*d*_*z*_ = 1.38, *p* = 0.001) and emotion expressiveness (*d*_*z*_ = 0.79, *p* = 0.03), and a non-significant small to medium effect on emotion knowledge (*d*_*z*_ = 0.58, *p* = 0.10) and emotion regulation (*d*_*z*_ = 0.44, *p* = 0.17). The results from the focus group suggest that parents and children enjoyed the program and that the program was useful and effective at impacting FMS and SEL.

**Conclusions:** This intervention is one of the first to intentionally target both FMS and SEL. Children, parents, and instructors deemed this program as acceptable and feasible. These preliminary findings warrant future evaluations of *Move 2 Smile*, including a randomized controlled trial.

## Introduction

Children today face at least three critical issues that can have long-term adverse effects on their health and development. Firstly, ~14% of children and adolescents are suffering from mental health problems, such as anxiety and depressive disorders, which can have a profound influence on well-being and social participation (1). Secondly, while 62% of children aged 3–4 years meet the Canadian Physical Activity Guidelines (2), after age 4, this drops precipitously, with only 35% of children aged 5–17 years meeting the recommended levels of physical activity [PA; (3)]. Finally, there is an increasing concern regarding children entering kindergarten without the necessary skills (e.g., self-regulation) to succeed (4). In fact, recent reports show that ~28% of children are deemed not ready for school when they arrive in kindergarten (5). All three of these issues are interrelated and have implications on success in the classroom, as well as on long-term physical and mental health outcomes as children get older (6–8). From an intervention perspective, it is critical to identify possible determinants that underlie all of these challenges in order to more effectively and efficiently intervene to improve the developmental outcomes for children and youth.

Two critical developmental domains that are hypothesized to underpin these broader issues are fundamental movement skills (FMS) and social emotional learning (SEL). With respect to motor development, the attainment of FMS such as running, jumping, and catching allows for a child to engage in more complex movement skills and, therefore, may increase opportunities for participation in PA (9). For example, a child must first learn how to throw a ball before engaging in the sport of baseball. Developing these fundamental skills at an early age allows children to independently engage in PA as they grow older and facilitates participation in recreational and organized games and activities (9). With respect to SEL, researchers are increasingly recognizing the importance of social–emotional skills such as self-discipline, emotion regulation, and motivation on mental health outcomes. Studies have shown that children who lack social–emotional skills are more likely to develop internalizing and externalizing problems and experience peer rejection, thus impacting overall mental health and well-being (10, 11). Furthermore, prosocial skills measured in kindergarten have been shown to significantly predict future academic success, such as completing high school, and are also correlated to substance abuse behaviors as an adult (12). There is emerging literature suggesting that FMS/PA behaviors and SEL/good mental health do not develop in isolation. A study conducted by Piek et al. (13) demonstrated that gross motor skill development from ages 4 months to 4 years was significantly related to anxiety and depression scores when children began kindergarten. Specifically, failure to attain specific motor milestones resulted in higher anxiety and depression in school-aged children. Furthermore, research has shown that through participation in PA, children can build friendships, learn how to resolve conflicts with peers, and develop self-advocacy skills (14). King-Dowling et al. (15) found that children aged 3–6 years with poor motor coordination also had co-occurring emotional and behavioral problems such as increased aggression and withdrawn symptoms. Wilson et al. (16) found that not only were motor skills significantly correlated with social skills and internalizing problems but also that social skills mediated the relationship between motor skills and internalizing problems.

Both FMS and SEL are considered key components of school readiness (17). Given the overlap of these concepts, it is clear that acquiring a broad set of social, emotional, and physical, particularly motor, skills prior to entering and during kindergarten provides a child with the best opportunity to succeed. In this sense, motor and social emotional skills are best viewed as determinants or factors that influence readiness to learn in the context of school. As such, it is critical to establish the building blocks of these domains in the early years in order to properly prepare a child to develop optimally as they enter the school system.

Given the interrelatedness between FMS and SEL and the impact they both have on mental health, PA, and school readiness, it would be logical to intentionally target these domains together in one program in preschool-aged children. While existing preschool interventions likely influence multiple domains of development, very few interventions are designed to intentionally target more than one aspect of development. Without specifically embedding activities that target domains of development that have been shown to be interconnected, these programs fail to address the complexity of the challenges our children face. Targeting multiple domains of development allow interventions to address larger, more complex issues like poor mental health, physical inactivity, and lack of school readiness.

Researchers in the INfant and Child Health (INCH) lab at McMaster University adopted a multicomponent approach and developed the *Move 2 Learn* program that targets motor and pre-literacy skills simultaneously (3, 18). This intervention runs for 10 weeks and each session is 60 min in length. Every session targets a different FMS and pre-literacy skill as well as includes time for free play. Parents participate in the program alongside their child and activities are designed for a parent–child dyad. The *Move 2 Learn* program resulted in significant improvements in both FMS and pre-literacy skills in children ages 3–4 years compared to a control group not participating in the program (3, 18). *Move 2 Learn* is evidence-based and is unique in several ways, including the involvement of parents, incorporating autonomous free play, and focusing on skills transferable in the classroom, such as turn-taking and listening during instructions. While this program was successful at impacting FMS and pre-literacy skills, both of which are key contributors to a child's development, it does not intentionally target aspects of SEL which are critical for addressing mental health issues. The modular structure of *Move 2 Learn* allows for flexibility in the content of this primarily movement-based program; therefore, it may be feasible to replace the reading component with activities aimed at teaching SEL.

The current study builds off of the *Move 2 Learn* program by introducing SEL into its program design and content. Activities aimed at developing SEL replace the reading skill component in *Move 2 Learn*. Capitalizing on the multicomponent structure of *Move 2 Learn*, this new program, *Move 2 Smile*, will target two of the core contributors of poor mental health, physical inactivity, and lack of school readiness: FMS and SEL. The primary objective of this study was to determine “proof-of-concept” as outlined by the ORBIT model for behavioral interventions in Phase IIA (19) by designing, implementing, and assessing the acceptability and feasibility of the *Move 2 Smile* program. The secondary objective was to examine the effect of this intervention on FMS and SEL in children ages 3–4 years. It was hypothesized that parents and children would rate the program sessions as enjoyable (4 or more on a five-point Likert scale) and the instructor would rate the sessions as feasible (4 or more on a five-point Likert scale). Although this study is exploratory in nature, it was hypothesized that children would improve their movement skill levels following the completion of the *Move 2 Smile* program (3, 18). It was also hypothesized that children's social–emotional skills would improve following the intervention (20, 21).

## Methods

### Study Design

This study was a mixed methods exploratory, feasibility study using a repeated measures, single-arm, within-subject design. This study was conducted across four phases: (1) recruitment and baseline testing, (2) *Move 2 Smile* intervention, (3) post-intervention testing, and (4) parent focus group. According to the ORBIT model for developing behavioral interventions proposed by Czajkowski et al. (19), this study falls within Phase IIA: Proof-of-Concept. The goal of this phase is to determine whether or not the intervention merits more rigorous and costly testing. Thus, it is recommended to use a within-subject design for interventions in this phase.

### Participants

Children and parents/primary caregivers were recruited through preexisting relationships between the INCH Lab and the University of Toronto Junior Blues Program along with recruitment across the Greater Toronto Area (GTA) in day cares, EarlyON centers (parenting support centers funded by the provincial government in Ontario), and community centers. In order to be eligible for this study, participants must (1) have been aged 3 years 0 months to 4 years 11 months, (2) not have had a preexisting intellectual or physical disability, and (3) have been free of any health condition (e.g., unstable heart condition) that would prevent safe participation in the intervention.

### Intervention

The intervention took place between February and April of 2019. Parents and children participated in each session jointly. The sessions were 60 min in duration and included three components: (1) direct movement skill instruction, (2) free play, and (3) SEL activities. See Table 1 for a weekly breakdown of the session activities. Each session was led by two Master's students, both of whom had experience working with children. In addition, six undergraduate students were trained as volunteers to help administer the program.

**Table 1:** Weekly skill breakdown of the Move 2 Smile program.

**Week**	**Motor skill**	**Social–emotional skill**
1	Balancing	**Prosocial behavior** and emotion expressiveness/regulation (dialogic reading)
2	Underhand rolling	**Problem solving**, emotion regulation (puppet role play)
3	Leaping and galloping	**Emotion regulation** (belly breathing with pinwheels, zones of regulation)
4	Underhand throwing	**Emotion knowledge** and emotion expressiveness (drawing faces)
5	Jumping	**Emotion knowledge** and emotion expressiveness (mix and match faces)
6	Overhand throwing	**Prosocial behavior** and emotion expressiveness/regulation (dialogic reading)
7	Catching	**Problem solving**, prosocial behavior, and emotion regulation (puppet role play)
8	Hopping	**Emotion regulation** (yoga with belly breathing, zones of regulation)
9	Kicking	**Emotion knowledge** and emotion expressiveness (emotion sorting game)
10	Striking	**Emotion knowledge** and emotion expressiveness (emotions bingo)

*Social–emotional skills in bold were the focus of that week. Social–emotional skills not shown in bold were the secondary skills targeted that week*.

#### Direct Movement Skill Instruction

The first component of the intervention (25 min) focused on movement skill development and was divided into four sections (warm-up, skill development block 1, skill development block 2, and an obstacle course). The intervention focused on three categories of FMS: balance, locomotor, and object manipulation skills. Each week focused on a different FMS and was taught using single-step skill acquisition strategies (i.e., introducing new skills one by one). This method of teaching FMS has been shown to elicit significant, positive changes in FMS in preschool-aged children (22). Movement skill activities progressed in difficulty each week, and within each session skill progressions were tailored to each child's comfort level and ability in order to create a mastery climate (23), suitable to the developmental stage of the children.

The first 5 min consisted of a whole body warm-up where the children and parents formed a circle and performed the “beanbag boogie” to music. The “beanbag boogie” is a children's song requiring children to move in different ways (e.g., march, run, or crawl) and balance a beanbag on different parts of your body (e.g., head, elbow, or stomach). The next 20 min consisted of two blocks of movement skill development and an obstacle course. During each movement block, the instructor would demonstrate a skill (e.g., catching) and then the parent/child dyads would spread out across the room to practice. Each block of skill development would progress in difficulty; for example, in the catching lesson, the parent/child dyads would begin with catching a big ball with two hands and then, in the second block, move to catching a small ball with two hands and then catching with one hand. During this time, the instructor and the volunteers would circulate around the room to help progress and regress the skills to maintain a mastery climate as well as provide tips to perform the skills better. After the two movement skill blocks, the children participated in an obstacle course that emphasized that week's skill combined with the skills they learned in the weeks prior. Children went through the obstacle course three to four times with their parents before moving on to the next component of the intervention.

#### Free Play

The next component of the intervention consisted of 10 min of unstructured free play (not involving the parent/guardian). The children were given access to a variety of different toys (blocks, puzzles, and equipment from the motor skill activities) to engage with, and no direct instruction took place during this time. The session instructor and the volunteers were asked to avoid initiating play with the child, but rather follow the child's lead.

#### Social Emotional Learning

The last component of the intervention consisted of 20 min of activities directly aimed at SEL. Following free play, the children and parents sat in a circle around the instructor to begin the SEL lesson. Depending on the activity, the children/parents would remain in the circle for a group lesson or the dyads would spread out across the room and complete the activities separately. In total, five components of SEL were targeted throughout the 10-week intervention: prosocial behavior, problem solving, emotion regulation, emotion knowledge, and emotion expressiveness. These components were chosen as they have been highlighted in the literature as key components of SEL that result in positive development across the life course (24, 25). See Table 1 for a complete breakdown of the weekly SEL activities. Each component of SEL targeted in the intervention was delivered over two non-consecutive sessions (e.g., week 1 and week 6) in order to ensure all children were exposed to each domain at least once should they miss two consecutive weeks. These modules were adapted from preexisting interventions targeting SEL (21, 26, 27).

#### Take Home Suggestions

At the end of each weekly session, the parents were provided with a one-page sheet that outlined the FMS and SEL activities that they performed during that session. This sheet included information on how to properly execute/teach the skills as well as some suggestions on how they could practice the activities at home. The parents were also sent home with any materials they used during the SEL component of the program. For example, the parents were allowed to keep the emotion bingo cards that were used in week 10.

### Outcome Measures

Outcome measures were chosen to assess the acceptability and feasibility of the *Move 2 Smile* intervention as rated by the children, parents, and instructors. Additionally, outcome measures were chosen to examine changes in motor, social, and emotional skills as a result of participating in *Move 2 Smile*.

#### Demographic Factors

A demographic questionnaire was administered to the parents at their baseline study appointment. The questionnaire included information regarding age, sex, race/ethnicity, parental education and occupation, and household income.

#### Acceptability and Feasibility

Acceptability was defined as both the enjoyment of the program as well as the level of parental engagement (attendance and participation in the take-home activities). Enjoyment was chosen as a measure of acceptability because it is a common feature in most behavior change theories as a key indicator of sustained engagement (28). The children and caregivers attending the sessions were asked to complete a questionnaire at the end of each session assessing the enjoyment and satisfaction with each activity on a five-point Likert scale, with higher scores indicating a more positive experience. A session leader completed a checklist following each session rating the feasibility of the activities and perceived enjoyment of the participants on a five-point Likert scale and provided general feedback. To measure parental engagement, attendance was taken at each session as a measure of adherence to the *Move 2 Smile* program. In addition, the parents/guardians who accompanied the child to the program were given a parental engagement questionnaire during the 10 min of free play that asked about how often they engaged in the take-home activities from the session before. The questionnaire had two sections: (1) FMS and (2) the SEL component. Each question asked about how often the parent/guardian practiced each activity with their child and is scored from 0 to 5 (0 = did not practice, 1 = one time this week, 2 = three times this week, 3 = every other day, 4 = daily, 5 = more than once a day). Each week, the parents received a total engagement score (average across all questions), an engagement score for motor (average of the motor questions), and an engagement score for SEL (average of the SEL questions).

#### Fundamental Movement Skills

FMS were measured using the Peabody Developmental Motor Scale 2nd Edition [PDMS-2; (29)]. The PDMS-2 is designed to measure both the fine and gross motor skills (GMS) of children from birth up until 6 years of age. For the purpose of this study, only the GMS subscales of the PDMS-2 were used given that the intervention targets FMS. The GMS subscales include stationary performance, locomotion, and object manipulation. A total score for each domain of GMS (stationary, locomotion, and object manipulation) is given by adding up the scores of each item in that domain (29). The PDMS-2 has a total of 143 items with a total possible score of 286. The stationary subscale consists of 30 items (total possible score = 60), the locomotion subscale consists of 89 items (total possible score = 178), and the object manipulation subscale consists of 24 items (total possible score = 48). The PDMS-2 has been previously established as a valid measure in 4-year-old children, with an inter-rater reliability of 0.89, and has been shown to be a valid measure to detect changes over time (29, 30).

#### Social–Emotional Learning

As recommended by Denham et al. (24), a battery of assessments were used to assess children's social and emotional competence from both the perspectives of the parent and direct child assessment. Emotional competence includes emotion expressiveness, emotional regulation, and emotional knowledge (31), and thus, all three of these skills were measured as part of the emotional competence measurement battery.

##### Emotion expressiveness and emotion regulation

The Social Skills Improvement System Rating Scales [SSiS-RS; (32)] was used to measure emotion expressiveness and emotion regulation along with other prosocial and problem behaviors. The SSiS-RS is a questionnaire reported by parents, teachers, and/or students regarding social and emotional behaviors. For the purpose of this study, only the parent version of the SSiS-RS was used. The tool comprised two rating scales that each have multiple subscales that measure social and emotional behaviors: (1) Social Skills (communication, cooperation, assertion, responsibility, empathy, engagement, and self-control) and (2) Problem Behaviors (externalizing, bullying, hyperactivity/inattention, internalizing, and autism spectrum). The coefficient alpha scores for the SSiS-RS parent form for the social skills and problem behaviors subscales are 0.96 and 0.94, respectively, supporting the internal consistency of this measure (33). In addition, the test–retest reliability for the parent version of the SSiS-RS ranged from 0.68 to 0.85 across all social skills subscales and ranged from 0.76 to 0.86 across the problem behaviors subscales. For the purpose of this study, the prosocial scale was used as a measure of social skills and the empathy and self-control subscales were further analyzed as a measure of emotion expressiveness and emotion regulation, respectively. The total raw scores for the prosocial scale (total possible score = 138) and the empathy (total possible score = 18) and self-control (total possible score = 21) subscales were used for analysis.

##### Emotional knowledge

The Affective Knowledge Test (AKT) was used to assess emotion knowledge. The AKT was specifically designed for preschool-aged children and uses printed faces as well as puppets in order to assess receptive knowledge, expressive knowledge, and situation knowledge (34). To measure expressive knowledge, the researcher pointed to each of the four faces (happy, sad, mad, and afraid) and the children were asked to verbally name that emotion. To measure receptive knowledge, the researcher would say an emotion and the child was required to nonverbally point to the correct face. Nine vignettes were enacted using puppets that were accompanied by visual and vocal cues performed by the researcher. Three of the nine vignettes represented stereotypical situations whereby the puppet would express the same emotion the child would typically experience in that situation (as indicated by the parent on a pre-assessment questionnaire). The remaining six vignettes represented non-stereotypical situations: the puppet would express the opposite emotion that the child would typically express in the same situation (again, indicated prior to the assessment). For example, if the parent indicated that the child would typically be scared of an approaching dog on the sidewalk, the puppet would act out happy when it saw a dog on the street. For each vignette, the child was required to point to the face that matched the puppet's emotion. For each item on the AKT, the child scored a 2 for identifying the correct emotion, 1 for identifying the wrong emotion but the correct valence (i.e., identifying scared instead of mad), and 0 for the wrong emotion and wrong valence. Scores were totaled to produce an overall score, receptive knowledge score, expressive knowledge score, and a situation knowledge score. The AKT has demonstrated both reliability and validity (35, 36).

### Procedure

Upon obtaining ethics approval, participants were recruited from families who attend local EarlyOn centers and preschools as well as those who attend Junior Blues programming. The participants were recruited using study flyers and recruitment material circulated via e-mail. Upon receiving a call/e-mail from interested families, the study information was e-mailed to the interested families. One week following, a follow-up call was made to determine eligibility, review the study information, and obtain verbal consent. Written consent was obtained at the first research appointment.

All participants completed a baseline appointment at the INCH Lab at the University of Toronto. At this study appointment, the parents/guardians were asked to complete the demographic questionnaire and the SSiS-RS, which took approximately 20 min. While the parents/guardians completed the questionnaires, the children completed the AKT and the PDMS-2 with a trained graduate student who had extensive experience administering standardized tests, including the PDMS-2 to preschool-aged children. After completion of all baseline appointments, the intervention was conducted over 10 consecutive weeks and all study participants attended the sessions with a parent or guardian. Additionally, an at-home component was introduced, where the parents and guardians were encouraged to complete target skill activities outside of the weekly sessions. Following the completion of the intervention, all the participants were asked to complete a follow-up appointment including the same assessments from the baseline appointment. All the participants completed their study appointments within 2 weeks from the start and end of the intervention. The study appointments took approximately 1 h to complete (10–15 min for the AKT and 30–45 min for the PDMS-2). In order to mitigate confirmation and performance bias, the intervention leaders were not involved in administering the assessments at baseline and post-intervention. The assessor was blinded to the performance of the child during the intervention sessions.

#### Parental Focus Group

Following the intervention, all the parents were asked to take part in a focus group to discuss the *Move 2 Smile* program and any feedback they may have. An interview guide was used to conduct the focus group and included questions pertaining to the acceptability of the intervention as well as changes in the physical and psychosocial domains that the parents observed (see Supplementary Appendix A: Interview Guide). One focus group was conducted by a trained graduate student who was not involved in the administration of the *Move 2 Smile* program. The focus group was audio recorded and transcribed verbatim.

### Statistical Analysis

The demographic characteristics were reported as means and standard deviations (SD). The median and interquartile range (IQR) were reported for the measures of acceptability and feasibility as these data were skewed. Descriptive statistics were computed on the ratings of each session as well as on the program ratings as a whole. These were further broken down into the two components of the program, FMS and SEL. Paired sample *t*-tests were used to examine changes in the FMS and SEL from pre- to post-intervention. Cohen's *d*_z_ was used as a measure of effect size (37). Cohen's *d*_*z*_ is an effect size calculation used for within-subject design whereby the effect size is calculated by dividing the mean difference of each measurement by the standard deviation of the difference scores (37). Based on the benchmarks suggested by Cohen (38), the effect sizes were interpreted as small (*d*_z_ = 0.2), medium (*d*_z_ = 0.5), and large (*d*_z_ = 0.8). All data were analyzed using SPSS Statistics version 21. For all analyses, significance was set at a two-tailed alpha value of 0.05.

### Qualitative Analysis

The focus group was audio recorded and transcribed verbatim. Thematic analysis was conducted to identify key themes and messages (39). All transcripts were first read over multiple times to familiarize the analyst with what transpired. Next, initial codes were produced by going through each section of the transcript individually. Pseudonyms were used for all participant names in order to maintain confidentiality. Once all segments of the transcript were coded, the analyst reviewed the codes and generated themes. All codes were then sorted into one of the themes. Lastly, the analyst reviewed all themes and labeled them appropriately.

## Results

### Participant Characteristics

Eleven families, including one family with a set of identical twin boys, were eligible and provided consent to participate in the study. One consented participant was deemed ineligible after the pre-assessment based on an apparent cognitive delay. While this participant was included in the program, they were not included in the final analysis. The final sample included 11 children (four girls) ranging from 36 to 59 months (*M* = 50.56, SD = 8.63). The demographic characteristics of the sample are shown in Table 2. Most of the parents had post-secondary education and four had graduate degrees.

**Table 2:** Participant characteristics.

	***M* (SD)**
Age (months)	50.56 (8.63)
	*n* (%)
Sex	
Male	7 (63.6)
Female	4 (35.4)
Child's ethnicity	
White	3 (27.3)
Chinese	2 (18.2)
Japanese	2 (18.2)
Other (mixed)	4 (36.4)
Parents' education	
High school	1 (9.1)
Some college	1 (9.1)
Some university	1 (9.1)
Bachelor's degree	4 (36.1)
Graduate degree	4 (36.1)
Parents' income	
Less than CA$50,000	3 (27.3)
Greater than CA$50,000	8 (72.7)
Previous programming	
Motor skill programming	6 (54.5)
SEL programming	3 (27.3)

*SEL, social–emotional learning*.

### Quantitative Results

#### Program Acceptability and Feasibility

The median attendance of the children and parents was 8/10 sessions (IQR = 1). The session ratings by the children and parents can be found in Table 3. The children and parents rated the sessions as enjoyable (>3/5) and rated the FMS and SEL activities similarly. Session enjoyment and feasibility as rated by the instructor can be found in Table 4. Much like the children and parents, the instructor also rated the sessions highly both in regard to enjoyment and feasibility. Parental engagement in the take-home activities is presented in Table 5. Across the 10-week intervention, the parents reported engaging in FMS practice about once per week (Med = 1.0, IQR = 0.3). In comparison, the parents reported engaging in SEL practice, on average across the 10-week intervention, about three times per week (Med = 2.3, IQR = 0.3).

**Table 3:** Enjoyment of each session as rated by the children and parents.

**Session no**.	**Child (enjoyment)**			**Parent (enjoyment)**		
	**FMS**	**SEL**	**Combined**	**FMS**	**SEL**	**Combined**
1	4.3 (0.6)	4.0 (1.5)	4.0 (1.0)	4.5 (0.8)	4.8 (0.9)	4.6 (0.8)
2	4.5 (0.3)	5.0 (2.0)	4.3 (0.8)	4.4 (1.8)	3.5 (2.5)	4.2 (2.0)
3	4.3 (0.5)	4.0 (1.4)	4.3 (0.6)	4.8 (1.0)	4.3 (1.3)	4.5 (1.0)
4	4.1 (0.4)	4.0 (1.0)	4.2 (0.3)	4.8 (0.5)	5.0 (1.0)	4.6 (0.6)
5	4.3 (0.5)	4.0 (1.3)	4.1 (0.6)	4.9 (0.9)	4.5 (0.8)	4.8 (0.8)
6	3.9 (0.8)	4.3 (1.8)	3.9 (0.7)	4.8 (0.5)	5.0 (.9)	4.8 (0.6)
7	4.4 (0.7)	3.5 (1.0)	3.8 (0.6)	4.8 (0.8)	4.5 (1.0)	4.7 (1.0)
8	4.5 (1.3)	5.0 (1.5)	4.3 (1.4)	5.0 (0.2)	5.0 (0.5)	5.0 (0.2)
9	4.0 (0.9)	4.8 (1.5)	4.0 (0.9)	5.0 (0.0)	5.0 (1.0)	4.8 (0.7)
10	4.4 (1.0)	4.0 (1.0)	4.0 (0.7)	4.6 (0.6)	4.5 (1.1)	4.5 (0.8)
Overall	4.3 (0.3)	4.0 (0.8)	4.1 (0.3)	4.8 (0.3)	4.6 (0.6)	4.6 (0.3)

*Results are presented as median (IQR)*.

*Combined = combined ratings from FMS and SEL*.

**Table 4:** Single instructor ratings of session enjoyment and feasibility.

**Session no**.	**Instructor (feasibility)**			**Instructor (enjoyment)**		
	**FMS**	**SEL**	**Combined**	**FMS**	**SEL**	**Combined**
1	3.5	4.0	3.6	4.0	3.5	3.8
2	4.3	3.0	4.0	4.0	3.0	3.8
3	4.3	4.5	4.3	4.3	4.0	4.2
4	4.8	4.0	4.6	4.3	5.0	4.4
5	4.5	5.0	4.7	4.3	5.0	4.5
6	5.0	5.0	5.0	5.0	5.0	5.0
7	4.8	4.0	4.5	4.8	4.5	4.7
8	5.0	5.0	5.0	5.0	5.0	5.0
9	5.0	5.0	5.0	5.0	5.0	5.0
10	4.5	5.0	4.7	4.5	5.0	4.7
Overall	4.6 (0.7)	4.8 (1.0)	4.7 (0.8)	4.4 (0.8)	5.0 (1.1)	4.6 (0.9)

*Results are presented as median. The overall results are presented as median (IQR)*.

**Table 5:** Weekly breakdown of parental engagement in take-home activities.

**Week**	**FMS**		**SEL**	
	**Med**	**IQR**	**Med**	**IQR**
1	1	2	1	3
2	0	1	3.5	3.5
3	1	1	2.3	4
4	1	1.5	2.2	2
5	1	1.2	2.6	1.4
6	0.7	1.5	2.2	1
7	0.7	1.6	2.5	0.7
8	0.9	1.5	2.3	0.7
9	1.2	1.2	2.3	1.7
10	0.8	1.4	2.2	1.8

*0 = did not practice this activity, 1 = one time this week, 2 = three times this week, 3 = every other day, 4 = daily, 5 = more than once a day*.

#### Changes in FMS and SEL

The results from the paired sample *t*-test analyses measuring the changes from pre- to post-intervention across FMS and SEL can be found in Table 6. Both FMS and SEL increased, on average, from pre- to post-intervention. There was a statistically significant improvement in total social skills and emotion expressiveness. The increases in FMS, emotion knowledge, and emotion regulation, however, failed to reach statistical significance. There was a medium to large effect size for both emotion expressiveness (*d*_*z*_ = 0.79) and emotion knowledge (*d*_*z*_ = 0.58) and a small to medium effect size for both emotion regulation (*d*_*z*_ = 0.44) and FMS (*d*_*z*_ = 0.42).

**Table 6:** Changes in FMS, social skills, emotion expressiveness, regulation, and knowledge.

	**Pre *M* (SD)**	**Post *M* (SD)**	***t***	***p***	**Effect size^a^**
Emotion knowledge	28.40 (4.77)	31.00 (2.26)	−1.85	0.098	0.58
Social skills	94.00 (8.94)	102.36 (3.05)	−4.57	0.001	1.38
Emotion expressiveness	13.09 (2.95)	14.82 (2.56)	−2.61	0.026	0.79
Emotion regulation	11.36 (2.16)	12.55 (2.38)	−1.46	0.174	0.44
FMS	239.1 (34.02)	246.73 (24.24)	−1.40	0.191	0.42

*FMS, fundamental movement skills*.

a *Effect sizes were determined using Cohen's d_z_*.

### Qualitative Results

The results from the focus group were sorted into 39 different codes. Those 39 codes were sorted into five themes, which are presented in Table 7.

**Table 7:** Summary of results from the thematic analysis of the focus group.

**Theme**	**Example quote**
1. Overall positive experience	“I thought like [the instructor] and the whole team were just really, really warm and friendly and welcoming and made everybody feel really comfortable.”
2. Structure of the program	“I felt that like there was maybe a little bit too much programming in that hour.”
3. Practicality of take-home activities	“I think there's too much paper and paper gets shoved places.”
4. Positive impact on SEL	“But in terms of development and the progression I think I found that they actually now can like say something about their feelings.for example, ‘Mommy, I'm going to be sad when you say something like that’ or something. Like they can understand what they are feeling right now.”
5. Positive impact on FMS/PA	“I did see incremental change every single week and by the end of it she was a little bit more gregarious, a little less cautious, a little bit more participatory.”

#### Acceptability and Feasibility

With regard to the enjoyment and feasibility of the program, two key themes were derived from speaking with parents: (1) an overall positive experience and (2) concerns regarding the structure of the program. An overarching theme throughout the entire focus group was that parents (and children) had an overall positive experience participating in the *Move 2 Smile* program. The parents agreed that the activities for both SEL and FMS were useful and applicable in everyday life. For example, speaking about the SEL activities, one parent stated that:

“[…] the belly breathing and zones of regulation for him was just huge. Like really, really helpful. And we use that with him at home and like he does it and it seems to work and be really effective.” (Paul, father of John)

Tara, the mother of Cassie, commented on the SEL activities, saying that, “through the program, you know, you kind of gave me a bit of a toolbox to work with in terms of pulling certain concepts like the zones of regulation.” Feedback was equally as positive for the usefulness of the FMS activities, Mark's (Tom's father) first thoughts about the program were, “I mean, overall I thought the, all the movement stuff was great, the ball throwing and the jumping and the balance stuff, it's all good stuff to go through, yeah.” In addition to the positive thoughts around the actual programming, the parents often mentioned the positive learning environment as one of the highlights of the program, for example:

“[…]the instructors made her feel comfortable right off the bat […] It was just like, so I think that came from feeling welcomed and supported because she wasn't like attached to me the whole time, so that was good.” (Penny, mother of Michaella)

Tara further commented:

“And I also really liked how all of the volunteers praised the kids for their effort as well, not for […] achieving […] the goal or whatever it was that you set out to do. But just even acknowledging the effort was fantastic.” (Tara, mother of Cassie)

It was evident that the instructors were a big part of the success of the program, and this highlights the importance of training for the instructors for future programs. As well, the mastery climate set by the program instructors appeared to be a key contributor to the success of the program. Paul summed it up nicely, saying,

“You can have the best programming in the world but like if you don't have like you know, really warm, like friendly people running it doesn't matter. But I thought like [the instructor] and the whole team were just really, really warm and friendly and welcoming and made everybody feel really comfortable.”

All the parents said that the children really enjoyed the program and they were sad that the program was over. Maureen said that, at the end of the program, her child, Kelsey, said, “Why can't I do it again, is there some more?”

#### Structure of the Program

A second key theme that came up when speaking about the quality of the program was its structure and the sessions themselves. The parents agreed that the SEL activities were too short, they seemed rushed, and that they would have liked to see more time spent on the activities. Maureen noted, “I felt that like there was maybe a little bit too much programming in that hour.” Sandra (mother of Alex) said, “But, yeah, the, I would say the emotions were, they were usually rushed.” Tara added:

“I would agree, I mean, I think you were really fighting against a lot of time constraints […] So by the end of it the social-emotional skills component did feel really, really, condensed […].” (Tara, mother of Cassie)

It was agreed upon that an extra 10–15 min added onto the session would be optimal according to the parents in order to allow more time for SEL activities. The parents disagreed about the free play portion of the program whereby some parents liked it and said that it provided a sort of “respite” between the FMS and SEL activities:

“[…] I kind of liked the free play because it offered a bit of an intermission or a respite between the two skill sets. So I think at least for my kid I think she really kind of appreciated that bit of a pause in the programming to let her do something relatively unstructured.” (Tara, mother of Cassie)

Some also liked that the free play mimicked what a school day would look like and helped prepare the child for what is to come:

“[…] once your kid starts school and stuff there's going to be recess and like similar, the equivalent sorts of things in their broader day schedule where there's a break and it's unstructured play… John didn't always like the free play, he wanted to be able to see me and stuff. But it's good for him to, I think I want him to work on just going, playing by himself.” (Paul, father of John)

On the contrary, other parents did not like free play and did not seem to understand why the free play was included in the program:

“And I understand the point of the free play was for us to sort of kind of just fill out our forms and questionnaires. But I, like Kelsey did not enjoy that. I think for my child, she needs structure, she needs focus and structure.” (Maureen, mother of Kelsey)

The parents had a few minor suggestions as to changes to the structure of the program that would have made the program better and more effective for them. However, overall, the parents were very pleased with the program and enjoyed their time in it.

#### Practicality of the Take-Home Activities

The practicality of the take-home activities was a third theme that arose in the parent focus group. While most parents agreed that the take-home activities were a nice addition to the program, it was evident that the handouts were not entirely feasible to implement at home. For example, one parent indicated that the paper handouts were not optimal:

“[…] I think there's too much paper and paper gets shoved places, like it just always does. If you ask me to find my papers from the program I wouldn't be able to.” (Penny, mother of Michaella)

Other parents agreed with this; for example, Tom's dad Mark said, “Yeah. I think it's that reminder thing because, honestly, once you leave on Saturday it's kind of gone.” and “…So a reminder during the week is, would be a great thing, yeah.” In addition to the impracticality of the paper handouts, some parents also expressed that they would have liked to have more physical aids for the movement activities similar to what they had for the SEL activities:

“But like the materials were usually focused on the emotion stuff. If there had been a material focused on the physical stuff […]. I think that would have helped me with the carryover.” (Penny, mother of Michaella)

The lack of physical aids may have been a reason for the lower levels of participation in the FMS take-home activities compared to the SEL activities. Overall, the feedback regarding the take-home activities not being entirely feasible is reflected in the low weekly engagement.

#### Changes in FMS/PA

Two important themes derived from the analysis of the focus group with parents were the positive impacts the program had on both FMS/PA and SEL. The parents were in agreement that the program was beneficial for developing FMS in the children, but also that they noticed changes in the interest and participation in active games. For example, one parent shared her observations of her child as a result of the program:

“I think overall the program for her was hugely beneficial. I did see incremental change every single week and by the end of it she was a little bit more gregarious, a little less cautious, a little bit more participatory […] I think she enjoyed it so much that she started to develop her own games that she's playing with us as well. So a lot of ball rolling, catching.” (Tara, mother of Cassie)

Another parent shared,

“[…] in terms of improvement I find she's really interested in balancing. She's always like, ‘Look at me on one foot, look at me on the other foot, look at me on the scooter, my foot is in the air.’ […] Well, she, we do the catching a lot more, like throwing balls cause like we just never thought that she'd be into it but she, from the program, that's an aspect.” (Penny, mother of Michaella)

It was evident that, according to the parents, the children became more interested in playing games and practicing FMS as a result of the program. In addition, the parents indicated that the children seemed more confident in their FMS following completion of the program.

#### Changes in SEL

With regard to SEL, the parents also noticed changes: “Socio-emotional for John was just huge. And that was one of the main reasons that we signed up, we were attracted to this program.” (Paul). One skill, in particular, that was mentioned was the ability to recognize and express emotions; one parent shared that

“[…] I think I found the, both kids actually now can like say something about their feeling, even in [family's first language], even in English. So, like, for example, ‘Mommy, I'm going to be sad when you say something like that’ or something. Like they can understand what they are feeling right now.” (Laura, mother of Tyler and David)

Another social–emotional skill that was brought up a lot in the focus group was the child's improvements in emotion regulation.

“And it was similarly in the middle of a tantrum that Cassie was having it was only when I said, ‘You are in the red zone right now, let's try and get back to the green zone’ that she started to kind of really, you know, calm down. It was, yeah, it was useful, very useful.” (Tara, mother of Cassie)

Another parent agreed and shared a similar experience:

“A while back John did something like, you know, broke something or whatever and then Tina was like, my wife's name is Tina, she said, ‘John, I'm really upset that you broke that.’ And he said, ‘Oh, mommy are you in the red zone?’ And he went through the whole thing. And then she said something like, ‘Yeah, I am.’ ‘What can I do to get you back in the green zone?’, he did the whole thing. ‘Maybe we should do some deep breathing.’ Like he said exactly the process that we said with him, yeah.” (Paul, father of John)

In addition to the changes in SEL parents saw in their children, they also highlighted the changes in themselves with respect to teaching SEL at home. Multiple parents shared the ways by which they have been able to apply SEL teaching strategies learned in *Move 2 Smile* into the home:

“Yeah, I would say it's sort of changed the way we read, I guess, like when we read I usually just would read them a book and he would ask me a few questions about the people. But now I'm more like, ‘What is that character feeling, like what would you feel if you were that character?’ like questions like that never occurred to me to ask after we read a story. So that helped.” (Sandra, mother of Alex)

Another parent shared how they applied SEL teaching strategies, saying that

“So I think, you know, she's been able to pause. Like it, sometimes it's really quick, like she likes to hit me and we have this book called Hands Are Not For Hitting. So it's always good to go back to that […]. So I guess, yeah, that's how we apply it.” (Penny, mother of Michaella)

Overall, the parents expressed that they felt their child's FMS and SEL improved as a result of the program. In addition, they felt as though the program has changed the way they foster SEL and FMS development at home.

## Discussion

The primary objective of this study was to assess the acceptability and feasibility of a program that targets both FMS and SEL in children aged 3–4 years. The results provide strong support for both the acceptability and feasibility of the *Move 2 Smile* intervention. Families attended the majority of the sessions, and the feedback collected after each session demonstrated that the children, parents, and instructors were all in agreement that the sessions were enjoyable. The results from the focus group further confirm the acceptability and feasibility of the program as the parents reiterated that the program was enjoyable and useful to their family.

It can be concluded, based on the high attendance rates (80%) and the positive session feedback from both the questionnaires and the focus group, that the program was highly acceptable to parents and children. Further, the instructor of the program rated the program as highly feasible to administer. This supports the feasibility of targeting two developmental domains together in one program during each session. This finding is positive given the important link between FMS and SEL established in the literature (13, 16). However, the participation rates of the take-home activities were lower than expected. On average, the parents engaged in the FMS activities once per week and the SEL activities three times per week. The participation in the FMS activities does not align with the results from the *Move 2 Learn* study (3, 18) whereby parents reported to engage in the at-home FMS activities at a rate of 46%, which equates to approximately 2–3 days a week. At-home participation was about the same for the SEL activities in the current study and the reading component of *Move 2 Learn* (3, 18). One explanation for this finding could be that families who enrolled in the *Move 2 Smile* program were more concerned with the social–emotional development of their child and therefore prioritized the SEL practice over the FMS practice. Feedback from the focus group alluded to this and also suggested that more physical aids for the motor skill practice may have encouraged more FMS practice at home. The take-home activities are an integral part of the *Move 2 Smile* program: they provide an important opportunity to practice the activities outside of the intervention sessions to further promote physical and psychosocial development. Given the low participation at home, specifically around the FMS activities, and the importance of this aspect of the program, steps should be taken to improve the acceptability and feasibility of the take-home activities. One suggestion that came out of the focus group was to provide the activities on a web-based platform or mobile app as the parents indicated that this would be more practical than the paper handouts. In addition, an orientation session could be included prior to the start of the program in order to explain to parents why the program is structured the way it is (i.e., combining FMS, free play, and SEL) and to provide information about the importance of practicing both the FMS and SEL activities at home.

In addition to examining the acceptability and feasibility of the *Move 2 Smile* intervention, this study also sought to examine the impact the intervention had on improving FMS and SEL. Overall, SEL had the most substantial improvements across the 10 weeks and the intervention had the largest effect on prosocial skills. In addition, parents' feedback in the focus group suggested a noticeable difference in social–emotional skills at home, such as regulating emotions. These results are in line with a similar intervention, Animal Fun, which is a motor-based intervention that also includes a SEL module for children 4–6 years old (20). The Animal Fun intervention resulted in a significant increase in prosocial skills in children who received the intervention, which aligns with the results of the present study. Interestingly, the current study had similar improvements in prosocial skills despite being delivered for only 20 min, once per week for 10 weeks compared to Animal Fun, which was delivered four times per week for a *minimum* of 10 weeks up to 6 months. In addition to prosocial behavior, emotion expressiveness was also shown to increase significantly after participating in the *Move 2 Smile* program, which is in line with current SEL interventions (21, 25, 26). However, there were no significant changes in emotion knowledge and regulation, which does not align with the results of the aforementioned studies. Posttest sensitization may have occurred, impacting the validity of the results reported by parents (40). It is possible that the parents rated their children lower following the intervention, having now had the chance to observe them in a classroom-like environment, something they may have never experienced prior, gaining knowledge related to aspects of SEL that would allow them to make more accurate ratings of their children's social–emotional skills post-intervention. It is important to note that emotion knowledge was assessed using the AKT as it is the most commonly used measure of emotional knowledge in preschoolers; however, researchers have noted a ceiling effect at 54 months of age (41). The mean age of the current sample was 50.56 months, so the children, not surprisingly, had high baseline scores on the AKT, which did not allow for much change. While some of the SEL measures did not reach statistical significance, as may have been expected based on similar studies, it is important to consider the duration of the *Move 2 Smile* program compared to other interventions. Similar studies that saw significant changes in SEL had programs ranging from 24 sessions to one academic year (25, 26). In contrast, *Move 2 Smile* was 10 sessions in length and only 20 min of each session was dedicated to SEL. Moving forward, it may be beneficial to increase the duration of the *Move 2 Smile* program and/or increase the SEL component from 20 to 30 min in order to increase the exposure and practice time.

With respect to the changes in FMS, the current study found a non-significant effect from pre- to post-intervention; however, the effect sizes ranged from small to medium. In addition, the parents' feedback in the focus group suggested that not only did they see improvements in their child's motor skills but also saw a greater interest and participation in physical activities. Given this, the possibility of important skill gain as a result of the intervention cannot be excluded. A general trend can be observed whereby participants typically increased in overall FMS across the two time points: 8 of 11 children (73%) scored higher following the intervention. An exception to this was that of three participants whose FMS decreased post-intervention. The aforementioned Animal Fun program primarily targeting FMS in young children did not elicit a significant change in motor skills immediately following the intervention (6 months), which aligns with the current study (42). However, the Animal Fun program did result in a significant change in motor skills from baseline to the 18-month follow-up assessment, suggesting a long-term improvement (42). The present study did not have a long-term follow-up assessment, and therefore a direct comparison cannot be made regarding the long-term effects of the program. Moreover, the changes in FMS in this study did not reach statistical significance, as seen with *Move 2 Learn* (3, 18). This was unexpected as the current study based its motor skill component of the program on *Move 2 Learn*. A possible explanation for this was that, due to the nature of the SEL activities in *Move 2 Smile*, the FMS portion of the program was cut down to 25 min compared to 30 min in *Move 2 Learn*, resulting in 50 fewer minutes of movement skill instruction over the 10 weeks. Further, parents in the *Move 2 Learn* study reported engaging in FMS practice at home 2–3 days per week compared to 1 day per week reported by the parents in the current study (3, 18). The higher rates of the at-home FMS practice reported in the *Move 2 Learn* study may explain the lack of FMS improvements in the current study. While both programs used the same manual for the FMS portion of the program, there was no measure of fidelity to the original program, and therefore it is also possible that the two programs were administered differently given the different program leaders. Moving forward, it may be important to keep the motor skill component at 30 min and extend the duration of the session by 10–15 min to account for both the FMS and SEL activities. Efforts should be made to increase practice at home through improving the feasibility of the take-home activities, specifically around the FMS activities. In addition, more training for session leaders and volunteers may improve outcomes.

## Limitations and Future Directions

The primary focus of this study was on assessing the feasibility and acceptability of the *Move 2 Smile* intervention. This study specifically sought out participant feedback regarding the delivery of the intervention and the specific activities included in the program, which is traditionally missing in extant literature. The ORBIT model recommends a small sample size for pilot studies in this phase (19); however, the small sample size rendered this study underpowered to detect changes in most of the secondary outcomes at a power of 0.80. While it was never expected for this study to be powered to detect changes in the secondary outcomes, it is difficult to ascertain the effectiveness of the *Move 2 Smile* program in improving FMS and SEL. Secondly, while a within-subject design is recommended for a pilot study, this study is limited without a control group to ascertain whether the changes in FMS and SEL truly arose from the intervention. Thirdly, the outcome assessors were aware that the children were participating in the intervention (not blinded) and the parents were not blinded to the purpose of the study (nor could they be given the design of the program, which requires active participation and informed consent), which leaves room for possible confirmation and detection bias by favoring more positive responses to the questionnaires following the study. The last limitation is regarding the measures administered to the child (PDMS-2 and AKT). Although both measures are deemed valid and reliable in preschool-aged children, behavioral issues may have impacted the results both in a positive and negative way. One child in particular was quite nervous at the pre-assessment and was evidently more comfortable at the post-assessment, and this could have contributed to the magnitude of positive change in FMS from pre- to post-intervention. Other children were less cooperative at the post-assessment, and this could have impacted their scores on the assessments. In addition to the behavioral issues, the AKT had a ceiling effect at 50 months, which may have contributed to the high baseline AKT scores and lack of significant change from pre- to post-intervention.

Despite these limitations, the results of this study demonstrate that the *Move 2 Smile* program is accepted by children and parents and is feasible to administer. Furthermore, the results demonstrate promising effect sizes for the changes in FMS and SEL. The results from this pilot study are encouraging and suggest that a multicomponent approach for a program like this can be beneficial; however, more work is needed. Firstly, this study warrants moving to the next phase of the ORBIT model, which is to conduct a larger, randomized pilot study using a control group to measure the true effects of the intervention. Before further testing, a few potential program modifications should be considered: (1) the session duration should increase to 70 min, allowing more time for SEL activities; (2) the SEL activities should include more peer-to-peer interaction; (3) the FMS activities should be delivered as intended for 30 min each session; and (4) alternative methods (e.g., a web-based platform) should be explored to increase the feasibility and participation in the take-home activities.

## Conclusions

This study is the first to explore the acceptability, feasibility, and preliminary effects of a multicomponent intervention targeting FMS and SEL in children under the age of 5 years. The results demonstrated that it is possible to target multiple domains of development together in one program, during each session, in preschool-aged children. This study demonstrated that a multicomponent intervention was accepted by the parents and children and was feasible to administer. Moreover, the combined results of the pre- and post-intervention assessments and the parent focus group suggested that the intervention may be positively impacting two key components of PA, mental health and school readiness: FMS and SEL. Given the impact of these developmental domains on current issues such as poor mental health and physical inactivity, this intervention has the potential to target more complex health concerns. By continuing to develop this program, *Move 2 Smile* may provide an acceptable and feasible multicomponent program that could be implemented in communities to have a positive impact on FMS, SEL, and development more broadly.

## Data Availability Statement

The raw data supporting the conclusions of this article will be made available by the authors, without undue reservation.

## Ethics Statement

The studies involving human participants were reviewed and approved by University of Toronto Research Ethics Board. Written informed consent to participate in this study was provided by the participants' legal guardian/next of kin.

## Author Contributions

MJ designed the study, coordinated recruitment and data collection, designed the social–emotional learning component of the intervention, co-lead the weekly implementation of the intervention, conducted the data analyses, and drafted the initial manuscript. CB assisted with the design of the study, data collection, data analysis, recruitment efforts, and revised and approved the final manuscript as submitted. EB designed the motor component of the intervention, assisted with data collection, and revised and approved the final manuscript as submitted. JC supervised the design and execution of all phases of the study and revised and approved the final manuscript as submitted. All authors approved the final manuscript as submitted and agreed to be accountable for all aspects of the work.

## Conflict of Interest

The authors declare that the research was conducted in the absence of any commercial or financial relationships that could be construed as a potential conflict of interest.

## Abbreviations

PA: physical activity
FMS: fundamental movement skills
SEL: social–emotional learning
GMS: gross motor skills
PDMS-2: Peabody Developmental Motor Scale−2nd Edition
SSiS-RS: Social Skills Improvement System Rating Scales
AKT: affective knowledge test
IQR: interquartile range.
