# Supplementary material for: The Acceptability and Feasibility of a Preschool Intervention Targeting Motor, Social, and Emotional Development
Source: Front Pediatr. 2020 Jul 10;8:319. doi: 10.3389/fped.2020.00319 (PMC7366389; doi:10.3389/fped.2020.00319)
Supplement: Supplementary file 1 [file Data_Sheet_1.PDF]

Appendix A: Interview Guide

1. **What was your first impression of the program?**

2. **How do you think your child felt about the program?**
   Probe: did they enjoy it?
   Probe: did they want to practice the activities at home?
   Probe: did they talk about the activities and the program?
   Probe: did they want to come to program each week?

3. **How did you feel about the program?**
   Probe: was the 10-week program a good length?
   Probe: how did you feel about the day and/or time of day the program was run?
      - how did you feel about the session length (1 hour)?

   Probe: how did you feel about being involved throughout each session?
   Probe: were the "Take Home" handouts easy to understand and follow?
   Probe: how did you feel about the activities we used in each session?

4. **How did the program meet or not meet your expectations?**

5. **How do you think the program has impacted your child's motor skills?**
   Probe: Have you seen improvements in their gross motor skills since the beginning of the program?
   Probe: What skills in particular have you seen improvements on?
   Probe: Do you think your child has become anymore physically active or interested in participating in physical games since the start of the program?

6. **How do you think the program has impacted your child's social and/or emotional skills?**
   Probe: Have you seen improvements in their social-emotional skills overall since the beginning of the program?
   Probe: What skills in particular have you seen improvements?
   Probe: How do you think this program has affected their behaviour at home and/or at daycare?
   Probe: Have you noticed any changes in how your child acts with other children?
   Probe: Have you noticed any changes in how your child acts around adults?

7. **What else might you have liked to see in the program?**

8. **Is there anything else you want to tell us about your experience in the program?**
